# Organotypic in vitro block culture model to investigate tissue-implant interface. An experimental study on pig mandible

**DOI:** 10.1007/s10856-021-06608-5

**Published:** 2021-10-28

**Authors:** Nagat Areid, Jaana Willberg, Ilkka Kangasniemi, Timo O. Närhi

**Affiliations:** 1 Department of Prosthetic Dentistry and Stomatognathic Physiology, Institute of Dentistry, University of Turku, Turku, Finland; 2 Department of Oral Pathology and Radiology, Institute of Dentistry, University of Turku, Turku, Finland; 3 Department of Pathology, Turku University Central Hospital, Turku, Finland; 4 Welfare Division, Oral Health Care, Turku, Finland; 5 Turku Clinical Biomaterials Centre (TCBC), University of Turku, Turku, Finland

## Abstract

In vitro studies of implant-tissue attachment are primarily based on two-dimensional cell culture models, which fail to replicate the three-dimensional native human oral mucosal tissue completely. Thus, the present study aimed to describe a novel tissue culture model using pig mandibular block including alveolar bone and gingival soft tissues to evaluate the tissue attachment to titanium implant provided with hydrothermally induced TiO_2_ coating. Tissue attachment on TiO_2_ coated and non-coated implants were compared. Ti-6Al-4V alloy posts were used to function as implants that were inserted in five pig mandibles. Implants were delivered with two different surface treatments, non-coated (NC) titanium and hydrothermal induced TiO_2_ coated surfaces (HT). The tissue-implant specimens were cultured at an air/liquid interface for 7 and 14 days. The tissue-implant interface was analyzed by histological and immunohistochemical stainings. The microscopic evaluation suggests that pig tissue explants established soft and hard tissue attachment to both implant surfaces. The epithelial cells appeared to attach to the coated implant. The epithelium adjacent to the implant abutment starts to change its phenotype during the early days of the healing process. New bone formation was seen within small pieces of bone in close contact with the coated implant. In conclusion, this in vitro model maintains the viability of pig tissue and allows histologically and immunohistochemically evaluate the tissue-implant interface. HT-induced TiO_2_ coating seems to have a favorable tissue response. Moreover, this organotypic tissue culture model is applicable for further studies with quantitative parameters to evaluate adhesion molecules present at the implant-tissue interface.

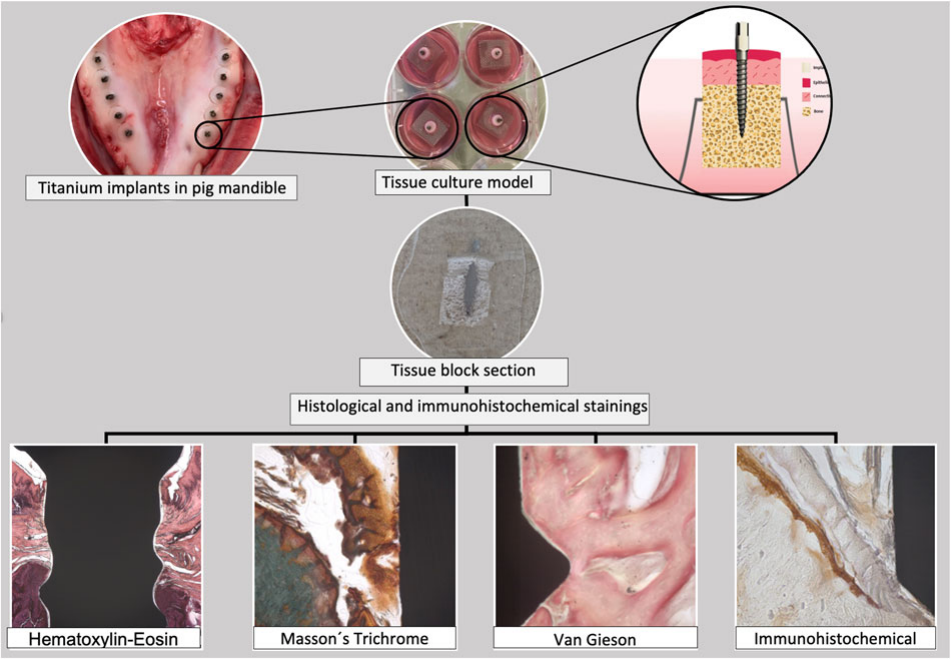

## Introduction

Besides the importance of sound and direct bone to implant contact, a proper soft tissue seal between the implant and the surrounding soft tissue is crucial for successful dental implants [1, 2]. The lack of a firm, soft tissue seal around the oral implant increases the opportunity for bacterial invasion, which may cause clinical complications such as inflammation, marginal bone resorption, and soft tissue recession leading to implant failure [3, 4]. Several studies have demonstrated that the non-keratinized peri-implant epithelium (PIE) cells attach to implant surfaces in a way similar to junctional epithelial (JE) cells, which attach to natural teeth through the basement membrane (internal basal lamina and hemidesmosomes) [5, 6]. However, this attachment is confined at the apical region of the PIE–titanium interface [6, 7]. Moreover, the implant lacks root cementum, and therefore the peri-implant connective tissue fibers are primarily oriented in a parallel direction with the implant surface [8]. The lack of these attachment structures on the implant surface weakens the biological seal around the implants and makes them more susceptible to bacterial invasion than natural teeth [9].

Nanoporous titanium dioxide (TiO_2_) coatings are examples of surface modifications that have shown good potential to promote soft tissue attachment on titanium surfaces [10–13]. In addition, hydrothermal treatment (HT) is an implant surface modification technique that has recently attracted attention for its ability to produce anatase crystalline TiO_2_ coating on titanium surfaces, improving their bioactivity and enabling the formation of a biologically active bone-like apatite layer on the titanium surface [14, 15]. The HT has been shown as a promising way to improve the bioactivity and osteoconductivity of the titanium implant [16, 17]. Previous studies by the authors have also demonstrated that the hydrothermal induced TiO_2_ coating promotes blood coagulation and encourages human gingival fibroblast attachment [18, 19]. However, little is known about their effect on the peri-implant soft tissue attachment.

Dental implant research focused on evaluating the soft tissue attachment to dental implant surfaces has relied mostly on animal models [11, 12, 20, 21]. The European Directive 2010/63/EU introduced the three R’s, Replace, Reduce, and Refine, limiting the use of animals for scientific purposes to the minimum [22]. The in vitro studies of implant-tissue attachment using cell culture models are primarily based on two-dimensional monolayer cell culture systems, which have several limitations. For example, they do not resemble the three-dimensional native human oral mucosal tissue. Furthermore, they are not suitable for testing final products that have different geometries and surfaces. Thus, the three-dimensional tissue-engineered oral mucosal model (3D OMM), which consists of both epithelium and connective tissue layers, has been modified to examine soft tissue/implant interfaces in vitro [23]. However, 3D OMM is technique-sensitive and demands good training to be used efficiently. The new tissue culture model developed in this study was modified from the previously reported rafted tissue culture model by Fukano et al. (2006) who introduced the skin tissue model to evaluate skin-percutaneous device interface [24]. More recently, Abdulmajeed et al. (2015) described an in-vitro tissue culture model using porcine gingival explants and experimental implants to evaluate soft tissue-implant interface [25]. The present study aimed to describe a novel tissue culture model using a mandibular pig block including alveolar bone and gingival soft tissues to evaluate the formation of peri-implant tissue attachment on an experimental titanium alloy implant provided with hydrothermally induced TiO_2_ coating. Tissue attachment on TiO_2_ coated and non-coated implants were compared.

## Materials and methods

### Implant preparation

A hydrothermal coating method (HT) was used to produce nanoporous titanium dioxide (TiO_2_) coatings on titanium surfaces. In this study, Surtex^®^ titanium (Ti-6Al-4V) endodontic posts (Dentatus Ltd, Stockholm, Sweden) were used to function as implants. The implants (posts) were polished using a polishing brush followed by pumice with cotton polishing buff wheels. Then they were cleaned ultrasonically with acetone and ethanol for 5 min. each and dried in the air before any surface treatments were carried out. Implants were delivered with two different surface treatments; machined non-coated (NC) titanium implants used as control samples and nanoporous TiO_2_ coated surfaces obtained by the (HT) coating method as described earlier [18]. In short, the hydrothermal suspension was prepared by dissolving titanium dioxide (TiO_2_), purified water, 1:10 diluted tetra methyl-ammonium hydroxide (TMAH) (N(CH_3_)_4_^+^OH)^−^, and mixed for 5 min. Titanium posts were laid at the bottom of Teflon containers consisting of a Teflon inner vessel and a stainless-steel jacket; within this, the hydrothermal suspension was added. Next, the vessel was kept at 150 ± 10 °C at a constant-temperature oven for 48 h. The titanium specimens were removed from the vessel and cooled in the air. The substrates were washed with distilled water in an ultrasonic bath for 10 min. All the specimens were sterilized in an autoclave (Tuttnauer Elara 11, Breda, Netherlands) for 20 min. at 121 °C and sealed in blister packages.

### Implantation and tissue culture model

**Fig. 1:**
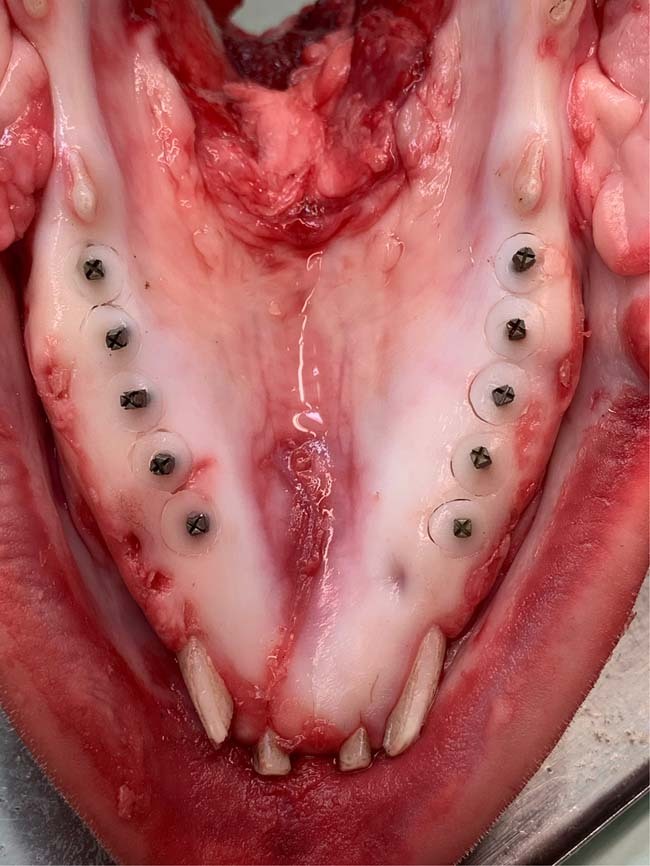
Titanium posts were inserted in freshly slaughtered pig mandible. The tissue/implant specimens were dissected using a 6 mm biopsy punch followed by a 6 mm Trephine bur

**Fig. 2:**
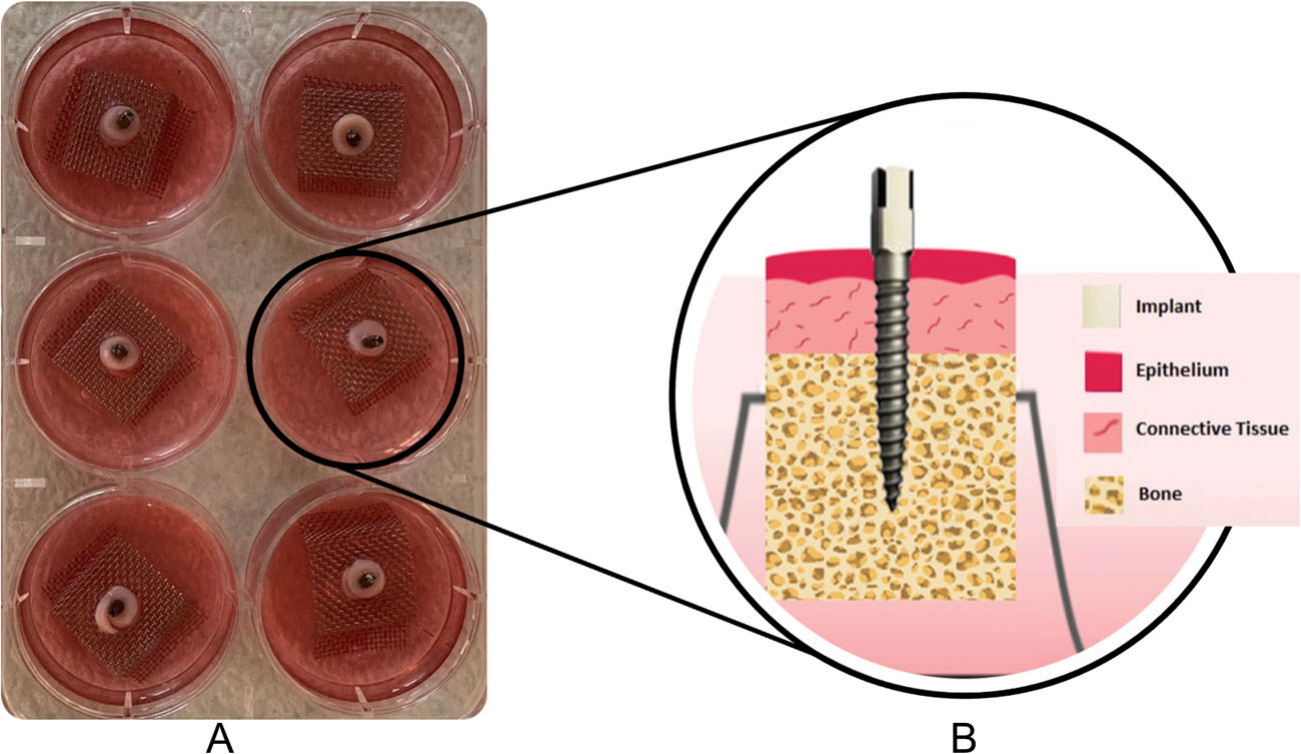
**A** The tissue/implant specimens were dissected and cultured in 6-well plates on a stainless-steel grid. The specimens were first soaked in the culture medium for two days; then, they were lifted to the air/liquid interface for 7 and 14 days. **B** Schematic view of the pig tissue culture model

Five mandibles were obtained directly from freshly slaughtered pigs. The experimental implants were inserted in the pig mandible with a length of 10 mm and a diameter of 1.35 mm (*n* = 40), using a self-tapping flapless technique. Then, the tissue/implant specimens were dissected using a 6 mm biopsy punch (Stiefel^®^ Biopsy Punch; Stiefel Laboratorium GmbH, Offenbach am Main, Germany), followed by 6 mm Trephine bur (Ulrich Storz GmbH, Tuttlingen, Germany) with water coolant (Fig. 1). The tissue/implant specimens were rinsed in PBS supplemented with penicillin, streptomycin, and amphotericin B. A total of 40 tissue/implant specimens (20 with HT treatment and 20 with NC surface) were then individually cultured on a stainless-steel grid, in 6-well plates containing Eagle’s minimum essential medium (EMEM M-2279; Sigma–Aldrich, St Louis, MO), supplemented with 10% fetal bovine serum (FBS), 100 U/Ig penicillin, streptomycin 100 Ig/ml, and 200 mM L-glutamine (Gibco BRL, Life Technologies, Paisley, UK). The specimens were first covered with the medium for two days. The culture medium level was reduced on day three, and the specimens were cultured at an air-liquid interface for two weeks (Fig. 2). The specimens were incubated at 37 °C in a 5% CO_2_ environment, and the culture medium was changed every day up to 7 and 14 days (*n* = 10/time point). Tissue specimens with no implants were also cultured to serve as baseline controls for general tissue morphology. At the end of each tissue culture time point, the specimens were placed in suitable embedding cassettes and fixed in 10% buffered formalin for one day at room temperature.

### Embedding of tissue culture samples

The embedding procedure used in this study was a modification of an early described embedding method using Technovit 9100 New^®^ polymerization system (Kulzer GmbH, Hanau, Germany) [26, 27]. Technovit 9100 New^®^ is a polymethyl methacrylate (PMMA)-based technical resin. It polymerizes in the absence of oxygen and at low temperatures (−2 to −20 °C), enabling different histological and immunohistochemical stainings for hard tissues. It is suitable for detecting the molecular composition of the implant-tissue interface [27, 28]. This embedding system involves different steps and components required to initiate the polymerization reaction, as illustrated in the Table 1, modifying the manufacturer’s protocol.

**Table 1:** A modification of the Technovit 9100 New^®^ embedding system used in this study illustrates the different solutions, steps, and incubation times used

The embedding steps	Solution	Temperature	Incubation time
Fixation	10% buffered formalin	(RT)	1 day
Dehydration	70% alcohol	(RT)	Overnight
	2 × 96% alcohol	(RT)	Overnight
	2 × 100% alcohol	(RT)	Overnight
	2 × xylene	(RT)	4 h each
Pre-infiltration solution 1	200 ml xylene + 200 ml stabilized BS	(RT)	Overnight
Pre-infiltration solution 2	l g dibenzoylperoxide (hardener 1) + 200 ml stabilized BS	(RT)	Overnight
Pre-infiltration solution 3	l g hardener 1 + 200 ml destabilized BS	+4 °C	Overnight
Infiltration solution	1 g hardener 1 + 200 ml destabilized BS + 20 g PMMA powder. (destabilized BS was added until the volume reached 250 ml).	+4 °C	Overnight
Polymerization		− 4 °C	2 days
Stock solution A	80 g PMMA + 400 ml destabilized BS + 3 g hardener 1. (more destabilized BS was added until the volume of the mixture reached 500 ml).		
Stock solution B	30 ml destabilized BS + 4 ml hardener 2 + 2 ml regulator. (more destabilized BS was added until the total volume reached 50 ml).		
	Solutions A and B are mixed in the proportion of (9:1)		

*PMMA* polymethyl methacrylate, *BS* base solution, *RT* room temperature

Following the fixation, the tissue/implant specimens were embedded in polymethyl methacrylate (PMMA) resin (Technovit 9100 New^®^ Kulzer GmbH, Hanau, Germany). This embedding procedure has been described previously [28]. In brief, the specimens were washed with running tap water for several hours and then dehydrated in a graded series of alcohol at room temperature overnight in the following steps; once in 70% alcohol, twice in 96% alcohol, twice in 100% alcohol, and then twice in xylene for 4 h. each at room temperature. The specimens were then pre-infiltrated overnight in pre-infiltration solution 1 followed by pre-infiltration solution 2, both at room temperature. After that, the specimens were incubated overnight in pre-infiltration solution 3, followed by the infiltration solution at +4 °C for both steps.

For the tissue polymerization step, each tissue/implant specimen was transferred from the embedding cassettes using plastic forceps and placed into the bottom of a precooled Teflon mold stored at +4 °C. Then the polymerization solution of a premixed 45 ml of stock A solution and 5 ml of stock B solution was added into the mold. The Teflon molds were placed in a vacuum desiccator cooled down to −4 °C. The specimens were then evacuated carefully in 200–400 mbar for 30 min. or to detect any gas bubbles on the surface. The desiccator was closed and stored at −4 °C, and the polymerization process was completed after 2 days. The hardened tissue blocks were pulled out of the molds and kept overnight under a lab fume hood for complete evaporation.

### Sectioning

Before the tissue block sectioning procedure, plastic slides were first glued onto the tissue blocks using a photocuring adhesive (Technovit 7210 VLC Kulzer GmbH, Hanau, Germany). The block surfaces were then ground using P800, P1200, and P2500 silicon carbide papers (Exakt Technologies, Oklahoma City, OK, USA). A glass slide was then roughened using a silicon carbide P800 paper (Exakt Technologies, Oklahoma City, OK, USA). One drop of Primer RC adhesion primer (Kulzer GmbH, Hanau, Germany) was placed on the glass slide to increase the bond between the resin and the ceramic surface. The tissue block was sandwiched against the glass slide with Technovit 7210VLC precision adhesive (Kulzer GmbH, Hanau, Germany) using a gluing machine (Exakt Technologies, Oklahoma City, OK, USA) with UV light for 15 min. The sandwich tissue blocks were sectioned into 100 μm thickness using a diamond band saw (Exakt Technologies, Oklahoma City, OK, USA). Then, the thickness was reduced to 20 μm by grinding the sections with silicon carbide papers of P500, P800, P1200, P2500 and polished with K4000 (Exakt Technologies, Oklahoma City, OK, USA). Ten specimens were sectioned per group (HT and NC) for each time point. Approximately 1–2 sections were obtained from each specimen.

### Histological analysis

Before histological stainings, methyl methacrylate-embedded sections were deplastinated by placing the section twice in xylene, twice in methoxyethyl acetate, twice in acetone, and twice in distilled water. Deplastinated sections were stained with Hematoxylin-Eosin, Van Gieson, and Masson Trichrome Goldner stains according to standard protocols. After stainings, the sections were rinsed in distilled water and dehydrated in a graded series of ethanol and xylene in the following steps; 70% ethanol, 96% ethanol, absolute ethanol, xylene, and then mounted with a rapid drying medium (Pertex, Histolab Products, Gothenburg, Sweden). The histological analysis of implant/tissue interface was carried out using a light microscope (Leitz Aristoplan, Leica Microsystems, Wetzlar, Germany). The images were captured using a digital camera (Leica DFC 320, Leica Microsystems, Wetzlar, Germany) and imaging software (Leica Application Suite version 4.1.0, Leica Microsystems, Wetzlar, Germany).

### Immunohistological analysis

For immunohistochemical staining, the methyl methacrylate-embedded sections were first deplastinated, as mentioned earlier. Antigen retrieval on PMMA sections was performed to detect the cytokeratin (CK) 14 protein. The sections were immersed in citrate buffer (pH 6.0) to perform heat-induced epitope retrieval for 10 min., followed by Tris-buffered saline (TBS) wash. The sections were then incubated at room temperature in a 3% hydrogen peroxide bath for 10 min. and washed again with TBS. After that, the samples were incubated for 30 min. at room temperature with Anti-Cytokeratin 14 antibody (1:30, BioGenex) and washed again in TBS. The sections were then incubated with Labeled polymer-HRP (Detection kit Envision + Dual-link system HRP (DAB+), Dako, Carpinteria, CA, USA) for 30 min. at room temperature. The TBS wash was repeated, and finally, the samples were incubated with diaminobenzidine (DAB) for 10 min. at room temperature, followed by an aqua wash. The sections were counterstained by placing them in hematoxylin for 3 min. at room temperature, and aqua wash was repeated. The samples were then blued with tap water followed with aqua wash and dehydrated in a graded series of ethanol and xylene in the following steps; 70% ethanol, 96% ethanol, absolute ethanol, xylene, and then mounted with the drying medium (Pertex, Histolab Products, Gothenburg, Sweden). The immunohistological stainings were analyzed as histological samples.

## Results

**Fig. 3:**
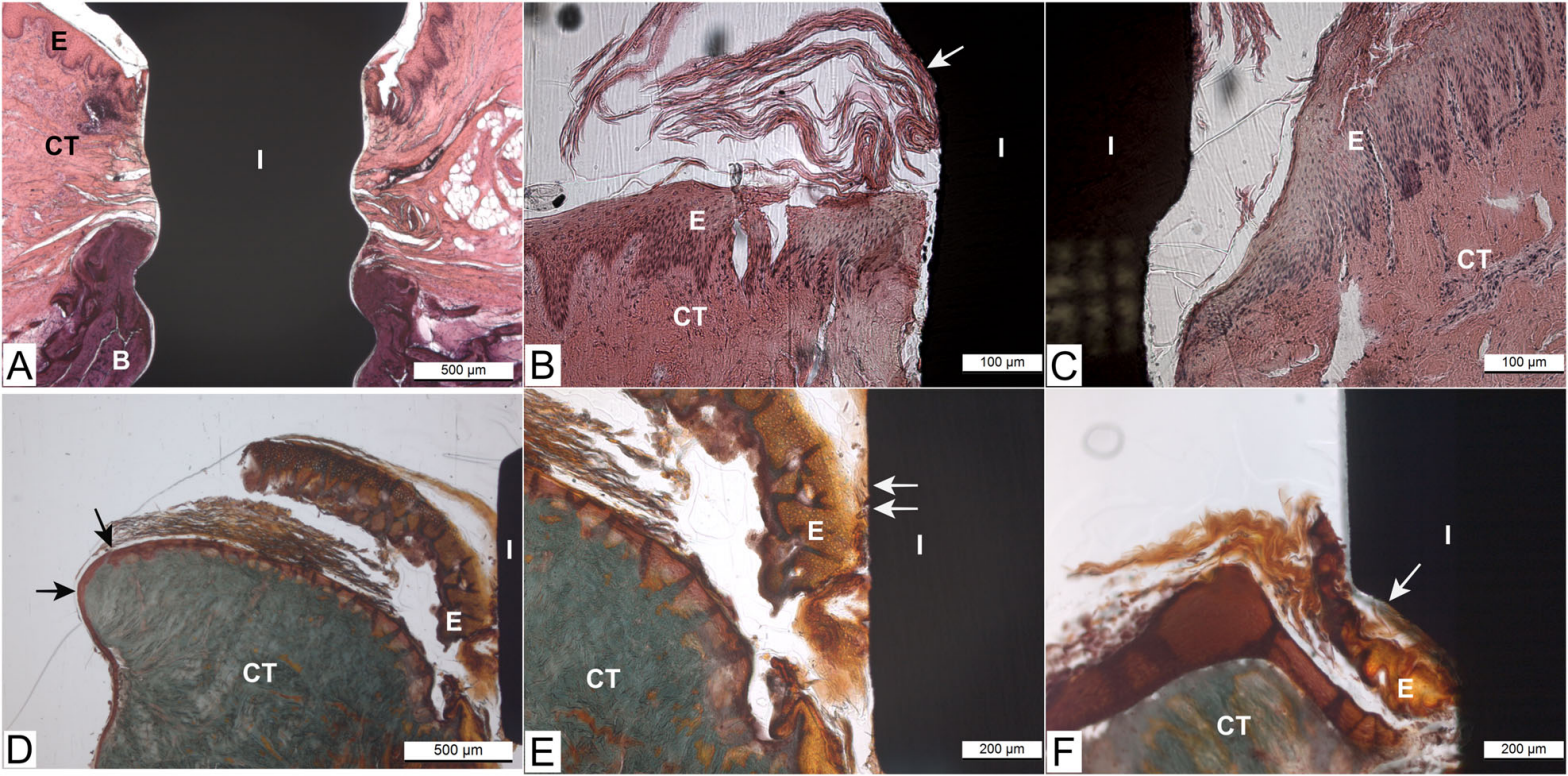
Light microscopy images of pig tissue/implant complexes cultured in vitro. Hematoxylin-Eosin stained sections of pig tissue explants attached to (**A**) and (**B**) hydrothermal (HT) coated and (**C**) non-coated implants at day 7 of culture. The epithelial, connective tissue and bone seemed to be in close contact with the coated implant surface. Despite the sloughing of the uppermost epithelial cell layers (a phenomenon that happened in the epithelium of all tissue explants), the upper part of the epithelium seemed to be attached to the coated implant surface (white arrow). Masson’s Trichrome stained sections of pig tissue explants attached to (**D**, **E**) HT TiO_2_ coated implant/tissue complex and (**F**) non-coated implants at day 14 of culture. Epithelial migration is visible on the side edges of the biopsy sample (black arrows (**D**)). White arrows indicate the epithelial attachment to the coated and non-coated implant surfaces (**E**, **F**). I implant, E epithelium, CT connective tissue, B bone

**Fig. 4:**
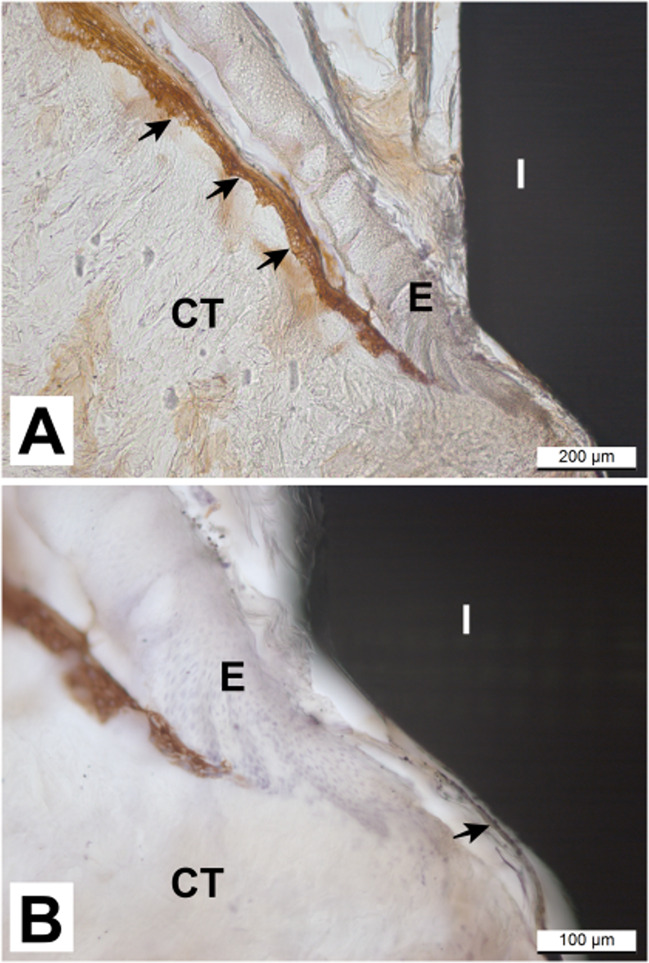
Immunohistochemical analysis of pig tissue/implant complexes at day 14 of culture using antibody directed against cytokeratin 14 (CK14) (**A**) non-coated implant/tissue complex. CK14 positive staining can be detected at the basal layers of the stratified gingival epithelium (black arrows). (**B**) HT TiO_2_ Coated implant/tissue complex. CK14 positive staining of basal layers of gingival epithelium ends ~200 µm apart from the implant surface. The black arrow shows faint positivity in the innermost cells facing the coated implant surface. There is a small gap between the epithelium and the implant because of the tissue cutting process

The results of this in vitro study showed that the overall structure of the pig tissue explants was intact and maintained throughout the culture period. Microscopic observation suggests that pig tissue explants showed epithelial, connective tissue, and bone tissue appeared attached to both implant surfaces (Fig. 3A through C). The epithelial cells of the pig tissue explants were seen to have migrated to cover the margins of the biopsy sample at day 14 of culture (Fig. 3D), suggesting that the epithelial cells were viable throughout the culture period and appeared to attach to the coated implant (Fig. 3B, E). The superficial layers of the epithelium, which start to slough off from the more basal layers during in vitro tissue culture, were attached to the implant surface (Fig. 3B through F). Immunohistochemical staining of CK14 showed positivity in the basal layers of the stratified gingival epithelium (Fig. 4A, B). The staining of basal layers ended a few hundred micrometers away from the implant surface. There was also some faint positivity in the innermost cells facing the coated implant surface (Fig. 4B).

**Fig. 5:**
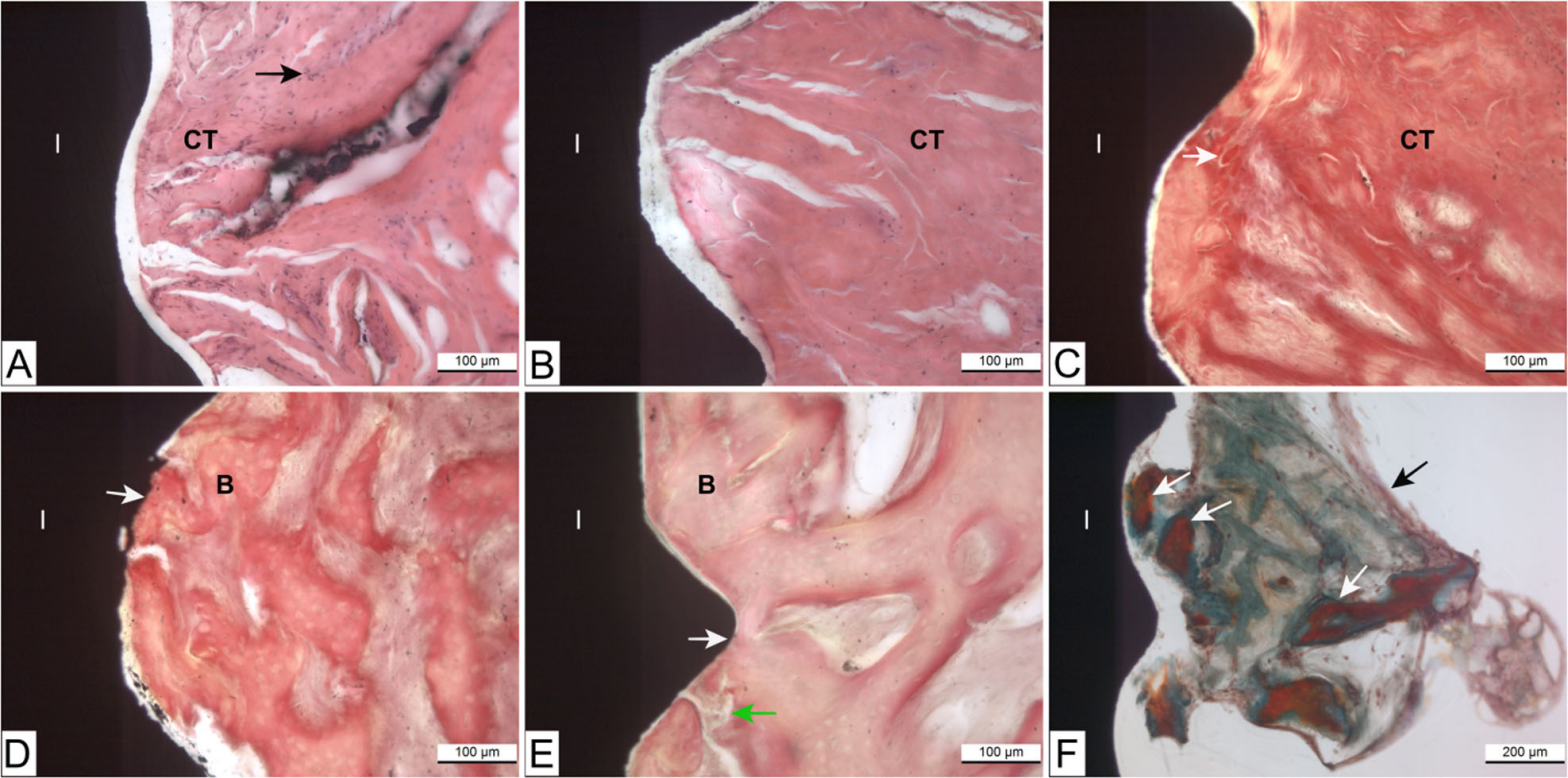
Light microscopy images of pig tissue/implant complexes cultured in vitro. Van Gieson -stained sections of pig tissue explants attached to (**A**) hydrothermal (HT) coated and (**B**) non-coated titanium implant at day 7 of culture. The connective tissue is adjacent to both implant surfaces. Several fibroblasts are detected along the coated implant surface (black arrow) (**C**) coated implant/tissue complex at day 14 of culture. The white arrow indicates thick collagen fibers (intense pink color). Both (**D**) coated and (**E**) non-coated implant surfaces are in close contact with the surrounding vital bone tissue at day 14 of culture (white arrows). The green arrow shows some tissue debris and coagulated blood between the implant and the bone. (**F**) Masson’s Trichrome-stained section of coated implant/tissue complex at day 14 of culture. White arrows indicate new bone formation in close contact with coated implant surface (orange staining on bone shows new bone formation). The black arrow shows migrated epithelial cells. NB. The gap between the tissues and the implant was an artifact because of tissue processing

Sections from day 7 of culture revealed that connective tissue was adjacent to both implant surfaces (Fig. 5A, B), with several fibroblasts detected along the coated implant surface. It seemed that after two weeks of culture, the collagen fiber organization had started. Some dense, thick collagen bundles were running parallel or slightly oblique to the coated implant surface (Fig. 5C). In the sections harvested at the 14 days of culture, both implant surfaces were histologically in close contact with the surrounding bone tissue (Fig. 5D, E). Some tissue debris and coagulated blood from the original bone were detected between the implant and the bone (Fig. 5E). New bone formation was seen within small bone pieces at the side edges of the tissue biopsy sample with the coated implant and in close contact with the epithelial cells derived from the gingival epithelium (Fig. 5F).

## Discussion

In the present in vitro study, we assessed the viability of the pig tissue model using mandibular blocks of freshly slaughtered pigs to evaluate the formation of peri-implant tissue attachment on TiO_2_ coated and non-coated implants. The pig model was used in this study because of its similarity to human periodontal tissue in terms of tissue structure, composition, and histological characteristics [29, 30]. Also, tissue culture models can be more informative than the conventional cell culture models and have economic advantages compared to the animal in vivo models. Tissue cultures are also easy to control since the biological processes that occur at the tissue implant interface are not disturbed by animal movements [25]. In this study, the overall structure of the pig tissue model was maintained throughout the culture period. The viability of tissues was also confirmed by the fact that the epithelial cells had migrated to cover the side edges of the biopsy sample. This was further supported by histological observations that epithelial, connective, and bone tissues were adjacent to both implant surfaces, revealing their good biocompatibility (Fig. 3A). At the implant-soft tissue interface, the creation of epithelial and connective tissue seals is essential to inhibit bacterial infiltration, prevent the development of peri-implant diseases, and ensure a long-term prognosis of dental implants [31]. The soft tissue seal around the dental implant is formed as a result of the wound healing process that starts immediately after the implant/abutment surgery when the blood proteins adsorb on the implant or abutment surfaces. This initial interaction may influence clot formation at the peri-implant wound site, which induces an inflammatory process and leads to tissue formation [20, 31–33]. The authors have previously demonstrated the potential of hydrothermal induced TiO_2_ coating to promote blood coagulation and consequently accelerate the wound healing process [18]. The findings of the current study are in line with a previous in vivo animal study describing the mucosal attachment to titanium implants [20], which showed the first signs of epithelial proliferation in specimens representing 1–2 weeks. The first weeks after implant placement is crucial for the formulation of the epithelial seal. Despite the uppermost layers of the epithelium starting to slough off from the more basal layers during the in vitro tissue culture, the epithelial cells seemed to be attached to the HT-coated implant surface. This finding provides evidence about the benefit of nanoporous TiO_2_ coating on PIE attachment.

PIE has its characteristic features in terms of ultrastructure and molecular composition, different from the junctional epithelium. A previous in vivo study by Fujiseki et al. (2003) investigated the immunohistochemical and the ultrastructural features of the PIE. They indicated that the PIE was structurally different from JE, showing slower cell proliferation, weaker expression of JE differentiation marker cytokeratin 19, and no evidence of true adhesion to the implant surface [34]. Many studies have investigated the phenotypic differentiation of the PIE [34, 35]. These studies reported that the staining pattern of cytokeratins of the PIE differed from that of the JE. However, there are not many studies describing the very early phases of PIE formation [32, 36].

In this study, an advanced embedding technique (Technovit 9100 New^®^) was used to describe the immunolocalization of the CK 14 protein. CK 14 is typically expressed by basal cells of stratified epithelium. In the in vitro model described here, CK14 was detected in the basal layers of pig gingival epithelium but not in the epithelium close to the implant surface, mimicking the PIE. It was also detected in the down-growing epithelium at the side edges of the tissue sample. The hypothesis is that the implant surface may influence the epithelial cells in a way that they start to change their phenotype into a more basal cell-like (mimicking JE/PIE). We also hypothesized that HT coating of the implant surface promotes soft tissue adhesion to the implant surface. These results are in line with a recent study by Roffel et al. (2019) evaluating the implant-soft tissue interface on a reconstructed human gingiva model [22]. They reported that the down-growing epithelium adjacent to the titanium abutment surface adapted its phenotype into a more basal cell-like and showed a specific immunoprofile resembling PIE. Cytokeratin 19, basement membrane proteins collagen IV, and laminin-332 were expressed between the epithelium and the hydrogel used in their model. Furthermore, a recent review investigating epithelium attachment to abutment surface showed that in the presence of the implant-abutment the epithelium starts to resemble a gingival margin, sulcular and junctional epithelium and express the associated physiological epithelial proteins and external and internal basement membrane proteins during the early days of the healing process [36]. The current study results using the advanced embedding method allow further studies of the precise immunoprofile of the developing PIE.

In addition, it is well known that the peri-implant connective tissue is characterized by more collagen fibers and fewer fibroblasts than the tissue around natural teeth [31]. Furthermore, the collagen fibers in the connective tissue around dental implants run parallel to the implant surface. Moreover, there is no evidence of insertion into the implant surface due to the implant’s lack of cementum and periodontal ligament [31]. In our study, under light microscopy, connective tissue attachment was seen on both implant surfaces. At two weeks of culture, the connective tissue collagen fibers along the implant-connective tissue attachment seemed more pronounced, with some thick collagen bundles running parallel or slightly oblique to the coated implant surface.

The incorporation of bone tissue in the model gives support and a basis for soft tissue reactions and enables the investigation of the association between the different cell types and tissues. It creates a real in vivo like atmosphere to study the primary formation of the soft and hard tissue attachment to the implant surface. Almela et al. (2018) developed an in vitro tissue engineering model consisting of hard and soft tissues based on primary cells isolated from oral tissues to mimic the natural structure of alveolar bone with an overlying oral mucosa [37]. They showed that this model could mimic the native oral tissues and act as an alternative to in vivo animal models.

Light-microscopic examination showed that both implant surfaces were in direct contact with the surrounding bone tissue. In the sections harvested at 14 days of culture, the osteoid formation was observed within small pieces of bone surrounded by gingival epithelial cells at the side edges of the tissue biopsy with the coated implant. This finding is in agreement with earlier in vivo works of Abrahamsson et al. (2004) and Berglundh et al. (2003) that described the early events of bone formation on titanium implant surface [38, 39]. Newly formed woven bone was detected on titanium implant surfaces at 1–2 weeks of healing, and it was considered to represent the first phase of osseointegration. The formation of new bone tissue in close contact with gingival epithelial cells opens an attractive new research area considering the interactions of epithelial and bone cells. The model described here allows the study of these interactions between different cell types.

Although the pig tissue model used in our current work includes all essential elements needed for in vitro studies of cellular and molecular interactions with dental implant/abutments, there are some limitations in the model that need to be assessed. Keeping the pig tissue explant intact and alive for prolonged culture time can be challenging. While this model revealed that the pig tissues survived throughout the culture period, soft tissue dehydration was seen in some histological images with longer cultures. Soaking the tissues in the culture medium for several days before lifting the cultures to the air-liquid interface may improve the tissue culture environment and prevent tissue dehydration. The culture medium used in our study supported the growth of different tissue types well. However, different cell culture supplements would possibly be needed for bone tissue and gingival soft tissue for longer culture. Due to the lack of blood flow, this in vitro model does not truly represent the wound healing biological process. However, it allows to evaluate the first events of peri-implant tissue healing. Considering that the thickness of the gingival tissue is uneven between different locations in the pig mandible, standardization of the implant positioning was not feasible. Therefore, the histomorphometric analysis was not performed. This study is a descriptive study based on histological observation with different classical histological stainings and immunohistochemical analysis. Light microscopy has limitations, including limited resolution and lower magnification compared to electron microscopy. In order to get optimum results with light microscopy and especially the analysis of immunostainings, the specimens need to be thin. In this study, the thickness of the sections was 20 μm, and only a few sections were obtained from one sample. The gap between the implant and the tissue is formed because of the technical challenges in cutting the delicate structure of hard-soft tissue attachment. Artifacts between these two different tissues can be easily produced, meaning that the preparation of one sample is technically demanding. However, the model presented allows for the investigation of the hard tissue-implant interface that is of crucial interest.

Earlier studies have shown that HT-induced TiO_2_ coated surfaces enhance surface wettability and improve human gingival fibroblast adhesion and proliferation, and at the same time, do not enhance bacterial adhesion and initial biofilm formation [19, 40]. Furthermore, in vivo animal studies using CaCl_2_ hydrothermally treated titanium implants have shown that HT treatment improved the PIE cells adhesion and reduced epithelial down-growth for a longer time post-implantation [41, 42]. Similarly, the present study suggests that an HT-induced nanoporous TiO_2_ coating seems to promote the formation of soft and hard tissue attachment on the titanium implant surface. However, a much larger number of samples are needed for a thorough evaluation.

## Conclusions

Hydrothermally induced TiO_2_ coating of titanium implants seems to enhance tissue attachment in a pig organotypic tissue culture model. The pig mandibular block tissue culture model used in this study is the first organotypic in vitro model that includes alveolar bone and gingival soft tissue elements and creates a real in vivo like atmosphere to study the primary formation of the soft and hard tissues attachment to the implant surface. This in vitro model maintains the viability of pig tissue and allows for histological and immunohistochemical evaluation of the tissue-implant interface. Moreover, it offers a time-efficient and inexpensive way for implant material studies that may reduce the need for animal experiments. Based on this work, the model described here is applicable for further studies with quantitative parameters to evaluate the tissue attachment and analyze the presence of adhesion molecules in the implant-tissue interface.
